# Seroprevalence of Canine Parvovirus in Dogs in Lusaka District, Zambia

**DOI:** 10.1155/2016/9781357

**Published:** 2016-09-06

**Authors:** Ngonda Saasa, King Shimumbo Nalubamba, Ethel M'kandawire, Joyce Siwila

**Affiliations:** ^1^Department of Disease Control, University of Zambia, School of Veterinary Medicine, P.O. Box 32379, 10101 Lusaka, Zambia; ^2^Department of Clinical Studies, University of Zambia, School of Veterinary Medicine, P.O. Box 32379, 10101 Lusaka, Zambia

## Abstract

Canine parvovirus (CPV) enteritis is a highly contagious enteric disease of young dogs. Limited studies have been done in Zambia to investigate the prevalence of CPV in dogs. Blood was collected from dogs from three veterinary clinics (clinic samples, *n* = 174) and one township of Lusaka (field samples, *n* = 56). Each dog's age, sex, breed, and vaccination status were recorded. A haemagglutination assay using pig erythrocytes and modified live parvovirus vaccine as the antigen was used. Antibodies to CPV were detected in 100% of dogs (unvaccinated or vaccinated). The titres ranged from 160 to 10240 with a median of 1280. Vaccinated dogs had significantly higher antibody titres compared to unvaccinated (*p* < 0.001). There was a significant difference in titres of clinic samples compared to field samples (*p* < 0.0001) but not within breed (*p* = 0.098) or sex (*p* = 0.572). Multiple regression analysis showed that only age and vaccination status were significant predictors of antibody titres. The presence of antibody in all dogs suggests that the CPV infection is ubiquitous and the disease is endemic, hence the need for research to determine the protection conferred by vaccination and natural exposure to the virus under local conditions.

## 1. Introduction

Canine parvovirus (CPV) is a major cause of morbidity and mortality in dogs worldwide. Infection with CPV results in a highly contagious enteric disease affecting mainly young naïve dogs or may result from vaccination failure due to maternal antibody interference [1]. Three antigenic variants, CPV-2a, CPV-2b, and CPV-2c, that differ by single amino acid residues of the VP2 capsid protein have so far been identified [1–3]. The clinical signs of CPV infection range from mild to severe foul-smelling haemorrhagic enteritis, fever, vomiting, and often death in severe cases [4]. Transmission of the parvovirus is most commonly through the faecal-oral route via contaminated food and water and the environment [5]. After being ingested, a viraemia develops with subsequent spread throughout the small intestines. The stability of the virus when shed in the environment promotes the spread through indirect transmission. Apart from domestic dogs, the virus has also been detected in several other species such as wild dogs and lions [6].

In Zambia, limited studies have been conducted to determine the prevalence of CPV in dogs. Only a single study found exposure of wild carnivores to CPV although no domestic dogs were examined [6]. There is also no study that has been conducted to evaluate the effectiveness of the vaccination or whether the dogs are protected or not. The majority of cases reported as being attributed to CPV by veterinary surgeons are based purely on clinical presentation since confirmatory diagnostic tests such as SNAP® tests and PCR are rarely done. Vaccination against CPV is routinely done using Vanguard Plus-CPV-2 strain NL-35-D vaccine (Pfizer) containing a monovalent modified live parvovirus which is given at 6 weeks of age. In addition, a multivalent preparation Vanguard Plus-5L containing canine distemper (CD) virus, canine adenovirus type 1 (CAV-1), canine adenovirus type 2 (CAV-2), canine parainfluenza (CPI) virus, canine parvovirus (CPV), and* Leptospira *antigens is also used.

The majority of dogs in high density communities are either free-roaming or semistray. These dogs receive minimal prophylactic or therapeutic veterinary care. The disease status of these free-roaming dogs is usually not known, nor is the history of previous exposure to common infections. Although the CPV cases in dogs (based on clinical presentation) have been reported in several veterinary clinics in Zambia, no seroprevalence study has yet been conducted to establish the extent of CPV exposure among dogs in Zambia. Therefore, the aim of this study was to provide information on the prevalence of antibodies to CPV in the dog population of Lusaka district in Zambia.

## 2. Materials and Methods

### 2.1. Study Area, Design, and Sampling

The samples used in this study were collected during a cross-sectional study conducted in Lusaka, to investigate filarial infections in dogs as previously described [7]. The samples were collected over a period of 5 months. Whole blood was collected in plain tubes for serum preparation from dogs aged six months and above that were presented for medical consultation at the School of Veterinary Medicine, University of Zambia (*n* = 111), or other nearby veterinary clinics (*n* = 63). Field samples (*n* = 56) from one of the townships of Lusaka were collected during an antirabies vaccination campaign. Consent to collect blood from the dogs was obtained from the owners after explaining the purpose of the study. Subject data was captured on a preprinted form.

Age was determined from owner's information and corroborated from dental examination when in doubt. The ages of all the dogs were then categorized as 1 (0–3 years), 2 (4–7 years), 3 (8–11 years), 4 (≥12 years), and 5 (adults of unknown age) because of the difficulty in determining the exact age of most of the subjects. There were equal numbers of unvaccinated (*n* = 115) and vaccinated (*n* = 115) dogs. The vaccinated dogs had received either a monovalent parvovirus vaccine or a multivalent vaccine. Vaccination status was obtained by a vaccination history and/or vaccination certificate. Other parameters collected included breed, sex, and source of subject (either clinic or field samples). The main outcome variable in the analysis was the presence of antibodies to canine parvovirus. The haemagglutination inhibition assay was used to determine the presence of antibodies specific to CPV.

### 2.2. Haemagglutination (HA) and Inhibition (HI) Assay

The HA test was carried out by preparing serial twofold dilutions of the modified live parvovirus vaccine (Vanguard Plus-CPV®) in 50 *μ*L of normal saline and 50 *μ*L of 1.0% fresh pig erythrocytes. The titre was expressed as the reciprocal of the highest dilution of haemagglutination. Newcastle disease Lasota vaccine virus strain was used as negative control antigen for the pig erythrocytes. The HI test was performed in 96-well microplates using 8 HA units of the parvovirus modified live vaccine virus. Twenty-five microlitres of the vaccine antigen was added to 25 *μ*L of twofold serial dilution of test serum and incubated for 1 hr at room temperature. Thereafter, 50 *μ*L of 1% pig erythrocytes suspension was added to all the wells, and the mixture was incubated in a refrigerator at 4°C. The reading of results was carried out after 1 hr and repeated after 24 hrs. The HI titre was expressed as the reciprocal of the highest dilution of serum showing complete inhibition of haemagglutination of pig erythrocytes [8].

### 2.3. Data Analysis

Data was entered into a Microsoft Excel® spreadsheet and examined for correctness and completeness. Descriptive statistical analysis and graphing was performed in R statistical software (R Core Team, 2014, Vienna, Austria). The analysis was performed after log transforming the antibody titre results. Statistical analysis was undertaken using a* t*-test for independent samples to determine the differences in the parvovirus antibody titres of unvaccinated and vaccinated dogs. Determination of predictors of antibody titres was performed using multiple linear regression models. The predictor variables analysed included source of subjects, sex, breed, and age (Table 1). Results were considered significant at *p* < 0.05.

## 3. Results

A total of 230 serum samples were collected comprising 130 male and 100 female dogs from four sources: 3 clinics (A; *n* = 9, B; *n* = 111, C; *n* = 54) and a field vaccination campaign (*n* = 56) (Table 1). The majority of the dogs (180/230; 78.3%) were of the mixed breed type while 50/230 (21.7%) were pure breeds (Boerboel, German shepherd, Jack Russell, Labrador retriever, Maltese poodles, Bull mastiff, Pomeranian, and Rottweiler).

**Figure 1:**
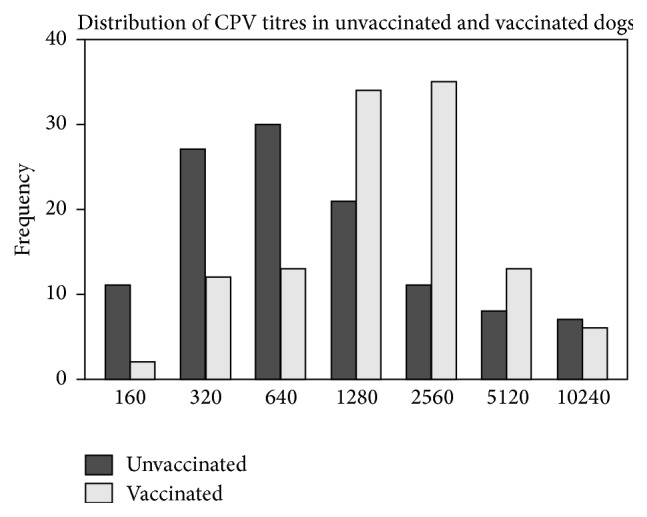
Distribution of CPV titres in dogs.

Seroprevalence in both unvaccinated and vaccinated dogs was 100%. The distribution of antibody titres ranged from 160 to 10240 (log = 2.2–4.0) with a median of 1280 (log = 3.1) (Figure 1). The mean titre for samples collected from dogs from veterinary clinics (clinic samples) was 2560 (log = 3.4) and that from the field sampling (vaccination campaign) was 640 (log = 2.8). The* t*-test showed a significant difference in antibody titres between unvaccinated and vaccinated dogs (*p* < 0.001) (Table 1). The analysis also showed that there was a significant difference in titres of dogs that were brought to the clinics compared to those that were sampled from the field vaccination (*p* < 0.000) (Table 2). No significant differences in antibody titres were seen between breed (*p* = 0.098) (Table 1) and sex (*p* = 0.572). A difference in titres among the age groups was also present but only between age groups 1 and 5 (*p* = 0.006) (Table 2).

A multiple regression model was performed to estimate the influence of the predictors (sex, breed, age, and source of the subjects) of the antibody titres. A significant model was developed for age and source of subjects (*F*-statistic: 13.29, adjusted *R* ^2^ = 0.097, df = 227, and *p* = 0.000).

## 4. Discussion

Canine parvovirus is a cause of morbidity and mortality in dogs, hence the need to investigate the prevalence of the antibodies to CPV. Although a few researchers have reported on CPV infection in Zambia [6], a number of putative cases are reported in veterinary clinics (Dr. Elizabeth Oparaocha, Showgrounds Vet clinic, personal communication). A number of these suspected cases are based on clinical presentation of haemorrhagic enteritis with absence of prior vaccination against CPV especially in young dogs less than six months of age [9]. At least one case of CPV infection per week is seen by clinicians in small animal practices (Dr. Andrew M. Phiri, UNZAVET Clinics, personal communication). In a study conducted in wild and domestic canids, CPV neutralizing antibodies were detected in lions and hyenas, but this was not done in dogs due to sample limitation [6].

The present study found that CPV is prevalent in Lusaka. All dogs, whether vaccinated or not, had anti-CPV antibodies indicating that they had been exposed to the virus. This finding is similar to previous studies in Zimbabwe and South Korea that found a high proportion of seroconversion in dogs [10, 11]. The antibody titres in dogs previously vaccinated were, however, significantly higher than those not known to have been previously vaccinated. In nonendemic areas or where compliance with vaccination is very high, natural exposure to CPV is low, thereby making the puppy series of vaccinations necessary.

The estimation of the contribution of the various predictors towards the antibody status of the dogs showed that age and vaccination status of the dog were the only significant predictors. The low coefficient of determination (*R* ^2^ = 0.10) of the model suggested that age and vaccination status could only account for a small variation in antibody titres and that there are many other factors besides vaccination and age that influence the observed titres of parvovirus in the dogs.

Whether a higher CPV antibody titre would lead to more superior protection against the disease or less severe clinical signs or both, resulting in better care outcomes, cannot be determined without carrying out challenge protection and neutralization tests. Although revaccination confers higher serum antibody titres and possibly protection against related strains of parvovirus, antibody titre levels may still need to be established before revaccination [12, 13]. The CPV antibody prevalence of 100%, although higher antibody titres were observed in vaccinated dogs, is evidence of the ubiquitous nature of CPV in Lusaka.

It was evident from this study that dogs that had received prior vaccination had significantly higher titres than dogs that did not have a history of vaccination (field samples). The differences in the antibody titres of dogs that were presented to the various clinics would suggest that these dogs received better or appropriate vaccination. The fact that these dogs were presented to the clinic by owners is an indication that such dogs had at one time most likely received a vaccine against parvovirus. In contrast, nearly all dogs that were sampled from the field antirabies vaccination campaign were not immunized against rabies or canine parvovirus. In endemic areas, dogs are generally exposed to the CPV in the environment and natural acquisition of protection may take place in which unvaccinated dogs eventually seroconvert [10, 14].

In conclusion, we found that CPV is endemic and exposure is common in unvaccinated dogs aged more than six months in the greater Lusaka region. Vaccination of dogs accounts for a small proportion (10%) to the relatively high CPV antibody titres observed in vaccinated dogs. Follow-up work to include serosurvey of dogs less than 6 months and comparing the *F*_1_ of vaccinated and unvaccinated dogs is recommended. A longitudinal study of antibody levels in puppies of vaccinated and unvaccinated dams until first vaccination at six weeks of age will be part of future studies.

## Figures and Tables

**Table 1:** Summary of the dogs sampled, vaccination status, age distribution (*n* = 230), and associated *p* values.

Variable	Number	Unvaccinated (%)	Vaccinated (%)	*p* value
*Source *				
Clinics	174	59	115	**<0.0001**
Field	56	56	0	

*Sex*				
Male	130	63	67	0.572
Female	100	52	48	

*Breed*				
Pure	50	11	39	0.098
Mixed	180	104	76	

*Site *				
A (clinic)	9	8 (89)	1 (11)	**0.001** ^*∗*^
B (clinic)	111	33 (30)	78 (70)	
C (clinic)	54	18 (33)	36 (67)	
D (field)	56	56 (100)	0 (0)	

*Age group (years)*				
1 (0–3)	163	85	78	**0.006** ^*∗*^
2 (4–7)	43	22	21	
3 (8–11)	6	4	2	
4 (≥12)	2	2	0	
5 (unknown)	16	2	14	

^*∗*^ANOVA.

**Table 2:** Pairwise comparison of antibodies titres of dogs in various clinics/sites, (*n* = 230).

Site	A	B	C
D (field)	*p* < 0.0001	*p* < 0.0001	*p* < 0.0001
A (clinic)	—	*p* = 1.000	*p* = 0.259
B (clinic)		—	*p* = 0.013
